# Early Childhood Outcomes After Neonatal Encephalopathy in Uganda: A Cohort Study

**DOI:** 10.1016/j.eclinm.2018.12.001

**Published:** 2018-12-20

**Authors:** Cally J. Tann, Emily L. Webb, Rachel Lassman, Julius Ssekyewa, Margaret Sewegaba, Margaret Musoke, Kathy Burgoine, Cornelia Hagmann, Eleanor Deane-Bowers, Kerstin Norman, Jack Milln, Jennifer J. Kurinczuk, Alison M. Elliott, Miriam Martinez-Biarge, Margaret Nakakeeto, Nicola J. Robertson, Frances M. Cowan

**Affiliations:** a Department of Infectious Disease Epidemiology, School of Hygiene and Tropical Medicine, London, UK; b Medical Research Council/Uganda Virus Research Institute and London School of Hygiene and Tropical Medicine Uganda Research Unit, Entebbe, Uganda; c Institute for Women's Health, University College London, London, UK; d MRC Tropical Epidemiology Group, London School of Hygiene and Tropical Medicine, London, UK; e Kyaninga Child Development Centre, Fort Portal, Uganda; f Mulago National Referral and Teaching Hospital, Kampala, Uganda; g Department of Paediatrics and Child Health, Mbale Regional Referral Hospital, and Mbale Clinical Research Institute, Mbale, Uganda; h University Children Hospital of Zurich and Children's Research Center, Zurich, Switzerland; i Homerton University Hospital, London, UK; j Uganda Maternal and Newborn Hub, Knowledge for Change, UK; k National Perinatal Epidemiology Unit, Nuffield Department of Population Health, University of Oxford, Oxford, UK; l Clinical Research Department, Infectious and Tropical Diseases, London School of Hygiene and Tropical Medicine, UK; m Department of Paediatrics, Imperial College London, London, UK; n Uganda Women's Health Initiative, Kampala, Uganda

**Keywords:** Neonatal encephalopathy, Outcomes, Neurodevelopment, Impairment, Cohort, Study, Uganda

## Abstract

**Background:**

Neonatal encephalopathy (NE) is a leading cause of global child mortality. Survivor outcomes in low-resource settings are poorly described. We present early childhood outcomes after NE in Uganda.

**Methods:**

We conducted a prospective cohort study of term-born infants with NE (n = 210) and a comparison group of term non-encephalopathic (non-NE) infants (n = 409), assessing neurodevelopmental impairment (NDI) and growth at 27–30 months. Relationships between early clinical parameters and later outcomes were summarised using risk ratios (RR).

**Findings:**

Mortality by 27–30 months was 40·3% after NE and 3·8% in non-NE infants. Impairment-free survival occurred in 41·6% after NE and 98·7% of non-NE infants. Amongst NE survivors, 29·3% had NDI including 19·0% with cerebral palsy (CP), commonly bilateral spastic CP (64%); 10·3% had global developmental delay (GDD) without CP. CP was frequently associated with childhood seizures, vision and hearing loss and mortality. NDI was commonly associated with undernutrition (44·1% Z-score < − 2) and microcephaly (32·4% Z-score < − 2). Motor function scores were reduced in NE survivors without CP/GDD compared to non-NE infants (median difference − 8·2 (95% confidence interval; − 13·0, − 3·7)). Neonatal clinical seizures (RR 4.1(2.0–8.7)), abnormalities on cranial ultrasound, (RR 7.0(3.8–16.3), nasogastric feeding at discharge (RR 3·6(2·1–6·1)), and small head circumference at one year (Z-score < − 2, RR 4·9(2·9–5·6)) increased the risk of NDI.

**Interpretation:**

In this sub-Saharan African population, death and neurodevelopmental disability after NE were common. CP was associated with sensorineural impairment, malnutrition, seizures and high mortality by 2 years. Early clinical parameters predicted impairment outcomes.

## Highlights

• This is, to our knowledge, the largest published cohort describing childhood outcomes after neonatal encephalopathy (NE) in sub-Saharan Africa
• Children with NE are at high-risk of multidomain neurodevelopmental impairment in a setting where access to supportive services are often lacking.
• Mortality is highest in the early neonatal period and in those with severe neurodevelopmental impairment
• Simple early clinical predictors may support a targeted approach to follow-up of particularly high-risk children

**Outstanding Questions:**

• What is the contribution of NE to cerebral palsy and other developmental disorders in low resource African settings?
• What strategies are effective to improve childhood outcomes in NE in way of prevention and intervention?
• What are the school-age outcomes after NE and later impacts across the life course?

**Research in context**

**Evidence before this study**

We searched Pubmed, and subsequent lists of relevant articles, without date or language restrictions, with combinations of the terms ‘neonatal encephalopathy’ (NE) ‘asphyxia neonatorum’, ‘birth asphyxia’, ‘perinatal asphyxia’, ‘hypoxic-ischaemic encephalopathy’, and ‘outcomes’, ‘neurodevelopment’, ‘disability’, and ‘impairment’ on Mar 25, 2018. NE has been estimated to affect 1·15 million infants around the world each year, including 478,000 in sub-Saharan Africa. A systematic review of long-term neurodevelopmental outcomes after intrauterine and neonatal insults showed one in five infants to be at risk of severe impairment in at least one domain; however, contributing data from low and middle-income countries (LMICs) were scarce. A pooled estimate of neonatal case fatality rates after NE in high mortality settings was reported as 27·7% (95% CI:18·7%–36·6%). Only two previous sub-Saharan studies (one each from Tanzania and South Africa) have reported impairment outcomes after NE. Both followed the NE survivors to ≤ 1 year, when impairment outcomes are less clearly defined and neither included a comparison cohort. The Tanzanian study reported impairment outcomes (defined as abnormal tone, convulsions, developmental delay or cerebral palsy) to only 6 months of age (n = 82). The second, from a tertiary South African centre, reported a prevalence of moderate–severe impairment of 33·3% after NE using a comprehensive neurodevelopmental assessment at one year of age, albeit amongst a small cohort of 36. We could find no studies reporting on later childhood outcomes amongst survivors of NE in sub-Saharan Africa.

**Added value of this study**

To our knowledge, this study is the largest cohort study to follow, comprehensively assess and describe early childhood outcomes amongst infants with NE in sub-Saharan Africa. We have documented high mortality to 27 months amongst children with NE, with most deaths occurring in the neonatal period. A third of survivors were affected by neurodevelopmental impairments, including cerebral palsy (CP) and severe global developmental disability without motor impairment. Bilateral spastic CP with dystonia was common and strongly associated with multi-domain impairment, visual and hearing loss, undernutrition and childhood seizures. Mortality at two years was high amongst children identified to have CP. NE survivors without CP or global developmental delay were still at increased risk of delayed fine and gross motor functioning. Simple neonatal clinical parameters associated with adverse outcome included severity of NE, presence of neonatal seizures, abnormalities on cranial ultrasound and persistent early feeding difficulties. A small head circumference at one year strongly predicted adverse outcome at two. Only four out of ten infants with NE experienced disability-free survival at two years of age.

**Implications of all the available evidence**

Children with NE in sub-Saharan African are at high risk of death and neurodevelopmental impairment with likely substantial impact on affected children and their families. Bilateral spastic CP, commonly associated with multi-domain impairments, seizures and malnutrition, affect many NE survivors in a region where access to supportive services are frequently lacking. Since studies examining the aetiology of CP often use a lower age cut-off of 2 years, the high burden of early mortality amongst children with severe impairment means that the contribution of NE to childhood disability is likely under-estimated. Simple early clinical predictors may support a targeted approach to follow-up of particularly high-risk children. Strategies for the prevention of NE, and early identification and intervention for those affected by impairment are needed to improve early childhood outcomes and quality of life for affected children and their caregivers. Current understanding of longer-term childhood outcomes of NE in resource poor settings is limited, but necessary if we are to understand the full impact of perinatal events on the life chances of affected children.

Alt-text: Unlabelled Box

## 1 Introduction

Neonatal encephalopathy (NE) is the third leading cause of under 5-year mortality and contributes substantially to long-term neurological morbidity worldwide [1]. Neonates affected by a perinatal insult typically present with NE, a descriptive term for a clinical constellation of neurological dysfunctions in the term infant [2]. Long-term sequelae amongst NE survivors include cerebral palsy (CP), global developmental delay (GDD), vision and hearing impairments and seizure disorders [3]. A systematic review of follow-up amongst high-risk newborns showed that one in five infants were at risk of severe impairment in at least one domain; however, few contributing data came from low and middle-income countries (LMICs) and in particular sub-Saharan Africa [4]. A common limitation of outcome studies amongst high-risk neonates is following to ≤ 1 year of age when neurodevelopmental impairment may be missed and diagnosis and classification of CP not possible [2].

Worldwide, 80% of the estimated 200 million children with physical and intellectual disability live in LMICs where implications for the health, wellbeing and life chances of affected individuals, families and communities are far-reaching [5], [6]. Early childhood development has been recognised as one of the pillars of the Global Strategy for Women's, Children's and Adolescents' Health, to ensure that children not only *survive*, but also *thrive* [7]. Children with GDD and other disabilities are vulnerable to health inequalities [8] and the social, emotional and financial impact on caregivers and other family members is high [9]. A focus on early child development is crucial to achieving the Sustainable Development Goals, to ensure that all children have the opportunity to maximise their full developmental potential and to improve life chances for themselves and their families [10].

The primary aim of our study was to determine the neurodevelopmental status of survivors of NE at 27–30 months of age in Uganda compared to a comparison cohort without NE and to describe the type and severity of neurodevelopmental impairment (NDI) in NE survivors. In addition, we aimed to describe nutritional status and identify clinical predictors for impairment outcomes after NE in this low-resource sub-Saharan African setting.

## 2 Methods

### 2.1 Setting

Uganda is a low-income country (LIC) with a neonatal mortality rate of 27 per 1000 live births [11]. Mulago National Referral Hospital in Kampala receives high-risk pregnancies from the surrounding areas. In 2012, more than 33,000 deliveries occurred on the low- (21%) and high-risk (79%) labour wards. In labour, fetal monitoring was by intermittent auscultation using a Pinard stethoscope and women were not routinely examined at the start of the second stage. Ventouse/forceps assisted deliveries were not routinely offered. A fifth of deliveries were by caesarean section (few are electively planned). Intravenous fluids, antibiotics and syntocinon were available. Midwife-led neonatal resuscitation included oxygen and bag-mask ventilation. Care on the 70-bed Special Care Baby Unit included simple continuous positive airway pressure ventilation, intravenous fluids including glucose (but not regular glucose monitoring), antibiotics and anti-seizure medication, but not mechanical ventilation, therapeutic hypothermia, cerebral function monitoring or brain imaging.

The study protocol was approved by the Uganda Virus Research Institute Research Ethics Committee, Mulago Ethics Committee, London School of Hygiene and Tropical Medicine, University College London and the Uganda National Council of Science and Technology.

#### 2.1.1 Study Design and participants

**Fig. 1:**
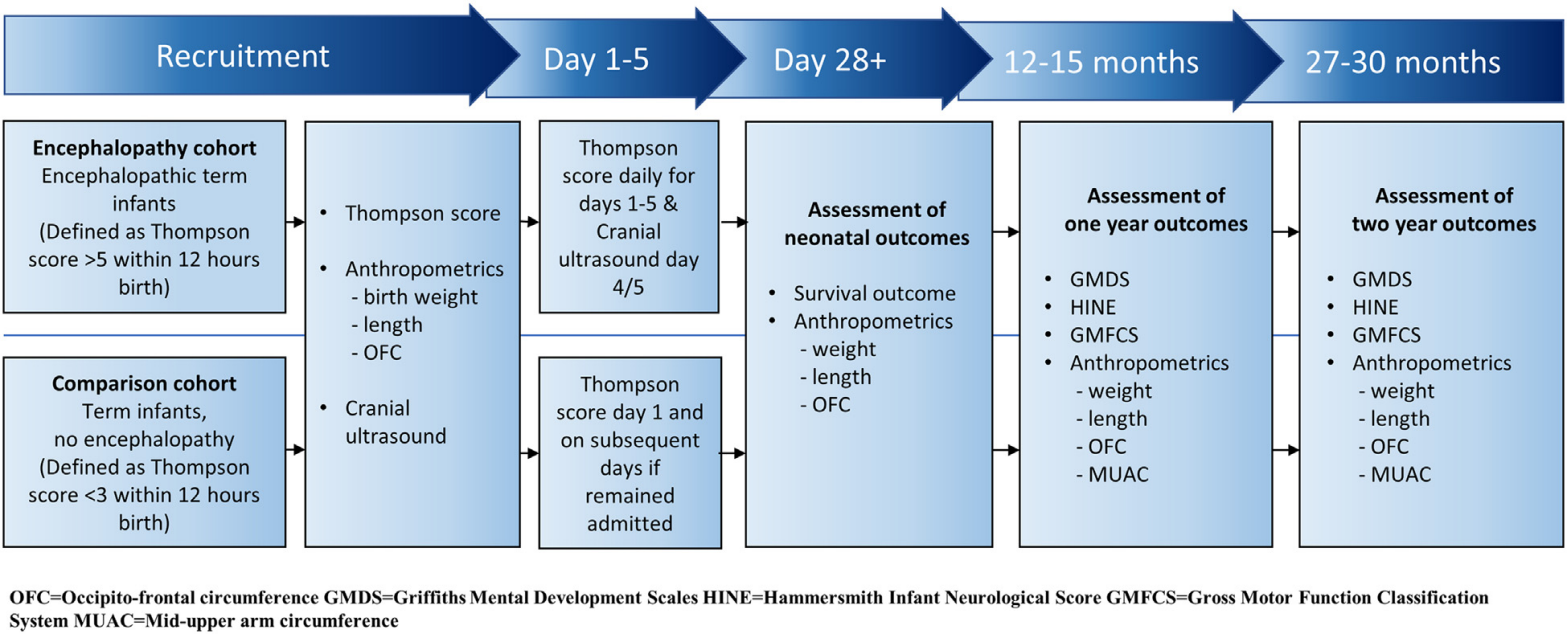
Diagram showing how participants moved through the study.

This study was a hospital-based prospective cohort study of neurodevelopmental outcomes at 27–30 months amongst term-born infants affected by NE and a contemporaneously recruited unmatched comparison group of term-born infants without NE (non-NE group). All participants were born at ≥ 37 weeks gestation and originally recruited to the ABAaNA case-control study [12] designed to investigate perinatal risk factors for NE in a low-resource African setting. NE was defined as a Thompson score [13] ≥ 6 within 12 h of birth [12]. For the controls, mothers and infants were systematically sampled from the labour ward admission book and were eligible for recruitment if their Thompson score was < 3 (Fig. 1).

Exclusion criteria included mother living > 20 km from the hospital (up to 20 km was deemed a reasonable distance for a new mother to travel for the 4–6 week postpartum study assessment), out-born infants, and no informed written consent. Infants with congenital abnormalities or other pathology were not excluded but a major anomaly was uncommon. Full study procedures and findings of the original case-control study have been described previously [12], [14], [15]. Socio-economic status was derived using principal components analysis of household characteristics and assets and categorised into tertiles.

#### 2.1.2 Measuring Clinical Predictors of Impairment Outcome

Encephalopathy was graded (mild, moderate or severe) from the most severe day (days 1–5) using a modified Sarnat classification [16]. Methods for measuring clinical predictors of outcome such as clinical seizures, hypothermia and neonatal serious bacterial infection have previously been described [14].

Cranial ultrasound scans (cUS) were performed on encephalopathic infants and the first 100 comparison children on recruitment (previously reported [15]) and again between days 3 and 5, using a portable machine (z.one ultra-Convertible Ultrasound System; Zonare Medical Systems Inc. Mountain View, California, USA). Images were anonymised and downloaded (OsiriX software, Geneva, Switzerland) and reported (by FC and CH), blinded to clinical data. The presence of recent and evolving injury was defined as clearly demarcated focal bilateral echogenicity in BGT and/or diffuse moderate, severe or dense echogenicity in WM [15].

#### 2.1.3 Follow-up Procedures

After discharge, families were contacted from comprehensive locator information collected at recruitment. Where a death was reported information was collected on date of death and parental report of causation. Surviving children were assessed at 4–6 weeks as part of the original study. Further funding was later awarded to see the children first at 12–15 months and then again at 27–30 months of age. Informed consent was taken individually at each of these visits. Transport costs were remunerated. Families not contactable by phone were visited at home (< 5%). Informed written consent from caregivers was obtained. This article focuses on neurodevelopmental and nutritional outcomes at 27–30 months.

#### 2.1.4 Neurodevelopmental Assessment at 27–30 Months

We used the Griffiths Mental Developmental Scales-II (GMDS) to derive an overall Development Quotient (DQ) from the six subscales (A to F) [17]. Assessors were trained study staff (MMB, FC, CT, RL, JS, KB, JM, EDB, KN) and all certified in GMDS and blind to presence of NE, all clinical history and imaging results.

All children were examined neurologically using a standardised scorable assessment, the Hammersmith Infant Neurological Examination (HINE), that has been validated as a predictor of motor outcome in different cohorts [18], [19]. The HINE is accessible, easy to perform and has good inter-observer reliability, even with inexperienced staff [18]. Optimal scores in term-born infants at 18 months are 75–78. A score of ≥ 67 at 9–14 months was predictive of independent walking at 2 years in a term-born cohort following hypoxic-ischaemic encephalopathy [20].

CP was diagnosed and classified according to the Surveillance of Cerebral Palsy in Europe hierarchical classification [21]; spastic bilateral, spastic unilateral, dyskinetic, dystonic, choreo-athetotic, ataxic or non-classifiable. CP severity was classified using the Gross Motor Function Classification System for Cerebral Palsy (GMFCS) [22]. Videos for all children with a suboptimal HINE score were reviewed by a minimum of two investigators (blind to NE status and other clinical data) with expertise in neurodevelopmental impairment (CT, FC and MMB) to type and classify the NDI. There was consensus between experts on impairment type and classification for all impaired children.

Neurodevelopmental impairment (NDI) was defined as a global DQ < 70 on GMDS and/or HINE score < 67 and/or diagnosis of CP. Poor outcome was defined as a composite of death or NDI at 27–30 months.

#### 2.1.5 Assessment of Hearing and Vision

Visual and hearing assessments were conducted according to HINE standardised procedures. In the HINE, intermittent or continuous deviation of the eyes or abnormal movements are noted as well as the ability to fix and follow on a clear black/white target; a hearing response is noted by a reaction to a stimulus (a rattle) held behind a visual range on each side. A score of < 1 (not following a visual target or not responding to an auditory stimulus) was used to define severe visual/hearing impairment.

#### 2.1.6 Anthropometrics and Health

Occipito-frontal head circumference (OFC, paper tape measure), weight, (SECA336 electronic scales, Hamburg, Germany) and height were taken by study staff using standardised procedures [23]. Haemoglobin (Hb) was determined on a finger prick sample using HemoCue Hb 201 (HemoCue AB, Angelholm, Sweden). Quality control was performed weekly following manufacturer's recommendation (HemoTrol, level 3). A structured maternal interview in Luganda reported on caregivers concerns regarding health, growth and development and episodes of illness including seizures and other neurological problems, feeding difficulties, chest infections, and treatment for malnutrition.

### 2.2 Statistical Analysis

The primary outcome for this analysis was NDI (a composite of GMDS, HINE and CP, as defined above) at > 2 years of age (27–30 months) when important outcomes, such as cerebral palsy, can be confidently diagnosed and classified. Secondary outcomes included GMDS and its subscales, HINE, CP, vision and hearing impairment, GDD without CP, and nutritional outcomes, all at 27–30 months. All-cause mortality to 30 months and poor outcome at 30 months (NDI or death, as described above) were also included as secondary outcomes. The primary comparisons were done between the NE cohort and non-NE children. Secondary comparisons were made between the same groups after excluding those with defined NDI from both groups.

We aimed to assess outcomes in at least 110 children with NE and 220 non-NE children, a sample size giving 80% power to detect a difference in mean DQ of 3.75 between the two groups, using Satterthwaite's *t*-test with unequal variances and assuming SD of 12 and 10 in exposed and unexposed cohorts, respectively [3].

Neonatal mortality was calculated as the percentage of neonatal deaths amongst those for whom vital status was known at 28 days. Post-neonatal mortality was calculated as the percentage of deaths amongst participants who survived to 28 days and for whom vital status was known at the end of 27 months of follow-up. Kaplan-Meier graphs were plotted, with children censored at loss to follow-up. Post-neonatal mortality was compared between NE and non-NE groups using chi-squared and log rank tests. Socio-demographic and baseline characteristics of participants were compared between NE and non-NE groups, using chi-squared tests and *t*-tests, as appropriate. Neurodevelopmental outcomes were compared between NE and non-NE groups; deviation from normality for GMDS and HINE scores meant median scores were calculated and compared using generalised Hodges-Lehmann median differences and 95% confidence intervals (CI), and the Wilcoxon rank sum test.

The proportion of children with neurodevelopmental, vision and hearing impairment at 27–30 months was calculated, with risk ratios and 95% CI using the non-NE cohort as the reference group, and P-values from chi-squared/Fisher's exact test. World Health Organisation data [24] were used to calculate weight-for-age and height-for-age Z-scores. OFC Z-scores were derived using the mean and SD from the non-NE group. Proportions with Z-scores < − 2 and − 3 were compared using risk ratios as above.

In secondary analyses, to assess whether outcomes after NE differed from the non-NE cohort, in the absence of defined NDI, analyses were repeated, comparing non-NDI encephalopathy survivors and non-NDI comparison cohort members. Clinical characteristics of children with NDI amongst both cohorts, including type and severity, were described.

The risk of poor outcome (death or NDI) and the risk of NDI amongst NE survivors were calculated according to severity of NE and other early clinical findings. Early clinical predictors of poor outcome were reported using risk ratios.

### 2.3 Role of the Funding Source

The study funders have no role in the study design, development or execution, data collection, analysis or interpretation, nor in the paper design, writing or decision to submit for publication. CJT had full access to all study data and final responsibility for the decision to submit for publication.

## 3 Results

Between September 2011 and October 2012, 210 infants were recruited to the NE cohort and 409 infants to the non-NE cohort. Characteristics of participants and predictors of NE identified in the original case-control study have been published [12].

#### 3.1.1 Follow Up at 27–30 Months

**Fig. 2:**
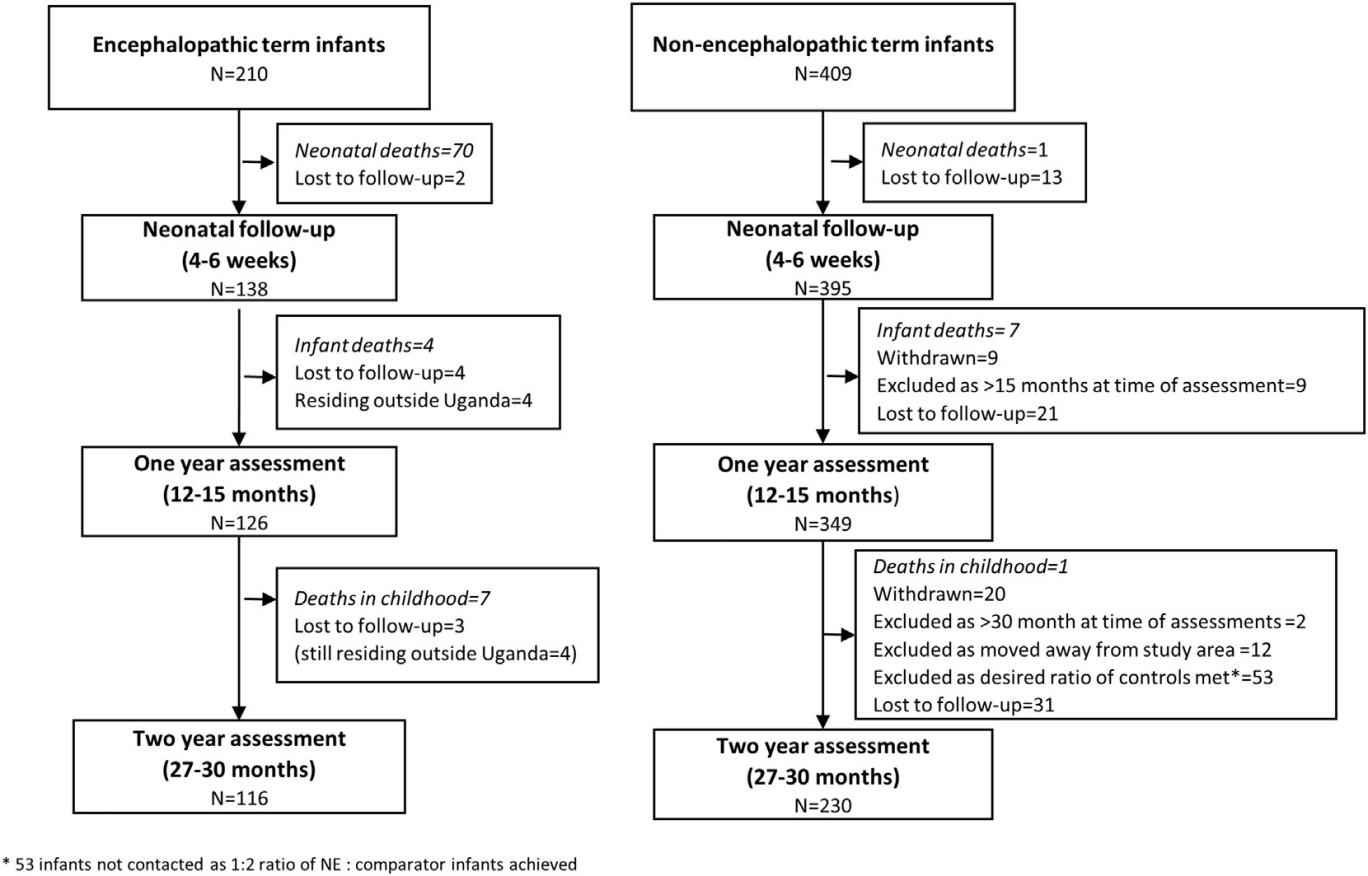
Flow diagram of participants.

Assessment at 27–30 months was achieved in 116 children with NE and 230 of the non-NE cohort. Details of the follow-up of the cohorts are given in Fig. 2. Thus, outcome data (known death or NDI outcome) was available for 93·8% (197/210) of the original NE cohort and 58·4% (239/409) of the original non-NE group; 71% allowing that 53 children were deliberately excluded when the desired follow-up number was achieved. Non-NE cohort mothers not seen at 27–30 months were, on average, younger, poorer, and primiparous and their infants were of lower birth weight than those that were followed (Supplementary Table).

Baseline and early clinical characteristics of the encephalopathy and non-NE groups seen at 27–30 months are shown in Table 1. Mothers of NE survivors were, on average, younger, more likely to be primiparous and less likely to be HIV positive than comparison mothers reflecting associations with NE reported in the original case-control study [12]. Encephalopathy survivors were more likely to be male, have a higher birth OFC, a poor 5-minute Apgar score, and to need resuscitation after birth. The majority had moderate or severe encephalopathy (82·8%) and almost half (48.3%) had clinical neonatal seizures.

**Table 1:** Baseline and clinical characteristics amongst neonatal encephalopathy (NE) survivors and the comparison, non-NE cohort - children seen at 27–30 months.

Clinical characteristic		Encephalopathy cohort N = 116 n (%)	Comparison cohort N = 230 n (%)	p-value⁎
Maternal Factors				
Socio-economic group (SES)	High	18 (15·7%)	49 (21·7%)	0·36
	Medium	78 (67·8%)	147 (65·0%)	
	Low	19 (16·5%)	30 (13·3%)	
Maternal age in years, mean (SD)		23·6 (5·2)	24·9 (5·4)	0·03
Maternal education ≤ primary school		41 (35·3%)	84 (36·7%)	0·81
Maternal primaparity		65 (56·0%)	84 (36·5%)	0·001
Maternal HIV positive		5 (4·3%)	27 (11·7%)	0·02
Emergency caesarean section		24 (20·9%)	38 (16·5%)	0·32
Infant factors				
Male sex		82 (70·7%)	114 (49·6%)	< 0·001
Birth weight in kg, mean (SD)		3·24 (0·44)	3·16 (0·46)	0·14
Birth occipito-frontal head circumference in cm, mean (SD)		35·5 (1·8)	34·9 (1·7)	0·004
Postnatal factors				
Apgar score at 5 min ≤ 5		39 (39·0%)	0 (0%)	< 0·001
Need for any resuscitation		87 (95·6%)	33 (15·1%)	< 0·001
Clinical features				
Grade of encephalopathy^a^	Mild	20 (17·2%)	–	
	Moderate	74 (63·8%)	–	
	Severe	22 (19·0%)	–	
Neonatal clinical seizures		56 (48·3%)	0 (0%)	< 0·001
Hypothermia: axillary temperature < 36·5C^b^		88 (77·2%)	52 (23·9%)	< 0·001
Hyperthermia: axillary temperature > 37·5C^b^		9 (7·8%)	4 (1·8%)	0·008
Haemoglobin in g/L, mean (SD)^b^		169 (26)	181 (20)	< 0·001

Missing data for SES (1 NE, 4 comparison), maternal education (1 comparison), Apgar scores (16 NE, 3 comparison), need for resuscitation (25 NE, 11 comparison), temperature (12 comparison), haemoglobin (30 comparison).

a Encephalopathy graded on the most severe day (days 1–5) according to Sarnat & Sarnat classification [16].

b Temperature and haemoglobin measured during day 1.

⁎ p-values calculated using chi-squared tests or Fisher's exact test for categorical data and t-tests for continuous data.

#### 3.1.2 Neurodevelopmental Impairment Outcomes After NE vs. Term Non-Encephalopathic Infants

Table 2 shows the neurodevelopmental and nutritional outcomes amongst NE survivors and the non-NE group. Nearly a third of NE survivors (29·3%) had NDI (compared to 1·3% of comparators); 19·0% had CP (all with other impairments) and 10·3% had GDD without CP. 16·4% of NE survivors either needed assistive devices for walking or were non-ambulant (GMFCS III-V). Amongst those with CP, median DQ was 31.1 (interquartile range (IQR): 5.1–50.0) compared to 60.0 (IQR: 53.1–66.1) for those with GDD without CP (p = 0·0015). Median HINE score was 41·5 (IQR: 20.1–49.1) and 76·3 (IQR: 73.3–77.8) for NE survivors with CP versus those with GDD without CP (p < 0·001). In the non-NE children, two developed mild CP, one GDD and one had an isolated hearing impairment. Amongst the 197 children with NE, disability-free survival occurred in only 41·6% (n = 82) of the original cohort. Fig. 4 shows the distribution of DQs for impaired survivors, unimpaired survivors and non-NE cohort. No child was categorised as being impaired based solely on sub-optimal HINE scores (< 67).

**Table 2:** Developmental and nutritional outcomes between neonatal encephalopathy (NE) survivors and the comparison cohort (non-NE term infants) at two years of age.

	Encephalopathy cohort (N = 116)	Comparison cohort (N = 230)	Median difference or RR (95% CI)^a^	p-value†
Developmental and neurology outcomes				
Griffiths Mental Development Scales				
Locomotor	84·5 (68·3, 106·8)	107·4 (89·5, 122·0)	-20·5 (− 27·4, − 13·9)	< 0·001
Personal social	106·2 (74·8, 116·7)	112·2 (102·1, 124·9)	− 11·3 (− 11·5, − 1·0)	< 0·001
Speech & hearing	85·3 (55·4, 88·2)	87·6 (83·0, 94·8)	− 7·4 (− 12·0, − 3·7)	< 0·001
Eye & hand	86·9 (64·8, 95·8)	99·7 (87·4, 110·1)	− 14·5 (− 19·1, − 9·0)	< 0·001
Performance	76·8 (64·7, 82·8)	81·5 (76·1, 85·0)	− 5·0 (− 7·6, − 2·8)	< 0·001
Practical reasoning^b^	90·4 (85·1, 99·6)	95·7 (87·5, 103·0)	− 3·7 (− 6·8, − 0·5)	0·02
Global developmental quotient (DQ)	90·4 (66·2, 97·5)	97·6 (91·1, 103·9)	− 9·4 (− 13·2, − 5·8)	< 0·001
Neurodevelopmental impairment (DQ < 70)	31 (26·8%)	1 (0·4%)	RR 61·5 (8·5, 444·6)	< 0·001
Hammersmith Infant Neurological Examination (HINE)^c^	77·8 (range 12·0, 78·0)	78·0 (range: 58·5, 78·0)	0 (− 0·5, 0)	< 0·001
Neurological score sub-optimal [18] (HINE < 67)	21 (18·1%)	1 (0·4%)	RR 40·9 (5·6, 300·4)	< 0·001
Vision				
Visual impairment, no NDI	0	0		0·004
Visual impairment, with NDI	5 (4·3%)	0		
Hearing				
Hearing impairment, no NDI	0	1 (0·4%)	RR 4·0 (0·4, 43·3)^d^	0·22
Hearing impairment, with NDI	2 (1·7%)	0		
Cerebral Palsy	22 (19·0%)	2 (0·9%)	RR 21·8 (5·2, 91·2)	< 0·001
Motor impairment: GMFCS				
Mild CP (GMFCS I-II)	3 (2·6%)	2 (0·9%)	RR 3·6 (0·6, 21·0)^e^	0·13
Severe CP (GMFCS III-V)	19 (16·4%)	0 (0%)		
Global developmental delay (GDD), no CP	12 (10·3%)	1 (0·4%)	RR 23·8 (3·1, 180·8)	< 0·001
Any neurodevelopmental impairment^f^	34 (29·3%)	3 (1·3%)	RR 22·5 (7·1, 71·6)	< 0·001
Childhood seizures^g^	15 (12·9%)	13 (5·7%)	RR 2·3 (1·1, 4·6)	0·02
Occipito-frontal head circumference (at 2 years)^h^				
Z-score < − 2 (< 45·8 cm)	16 (13·8%)	7 (3·0%)	RR 4·5 (1·9, 10·7)	< 0·001
Z-score < − 3 (< 44·3 cm)	10 (8·6%)	0		< 0·001
Any neurological sequelae including CP, GDD, profound visual or hearing loss or childhood seizures	39 (33·6%)	17 (7·4%)	RR 4·8 (2·8, 8·3)	< 0·001

Nutritional outcomes				
Moderate under-nutrition: weight-for-age Z-score < − 2	18 (15·6%)	19 (8·3%)	RR 1·9 (1·0, 3·4)	0·04
Severe under-nutrition: weight-for-age Z-score < − 3	12 (10·3%)	3 (1·3%)	RR 7·9 (2·3, 27·6)	< 0·001
Wasting: weight-for-height Z-score < − 2^i^	8 (6·9%)	11 (4·8%)	RR 1·4 (0·6, 3·5)	0·42
Stunting: height-for-age Z-score < − 2^i^	33 (28·4%)	45 (19·7%)	RR 1·4 (1·0, 2·1)	0·06
Anaemia				
Haemoglobin (Hb), mean (SD)	10·5 (1·4)	10·8 (1·3)	− 0·3 (− 0·6, 0·0)	0·03
Anaemia (Hb < 11·0 g/dL)	72 (62·1%)	130 (56·5%)	RR 1·1 (0·9, 1·3)	0·32

Data shown are median (IQR) for continuous data unless otherwise indicated, and n (%) for categorical data. CP = Cerebral palsy, DQ = Developmental quotient, GDD = Global developmental delay, NDI = Neurodevelopmental impairment, RR = risk ratio.

a Generalised Hodges-Lehmann median differences for continuous data and RR for categorical data for the encephalopathy cohort using the comparison cohort as the reference group.

b Not available for 31 encephalopathic survivors for whom the global DQ was calculated as the mean of the remaining five subscales.

c Not done for 4 children in the comparison cohort.

d RR calculated for any hearing impairment versus none.

e RR calculated for GMFCS I-II (milder impairment) versus GMFCS = 0. RR could not be calculated for GMFCS III-V (severe impairment).

f Neurodevelopmental impairment: global DQ < 70 and/or HINE < 67 and/or diagnosis of CP.

g Defined as seizures outside the neonatal period.

h Cut-offs for head circumference was determined as comparison group mean – 2 comparison group SD and mean – 3 comparison SD.

i Not available for 1 child in the comparison cohort.

† p-value from Wilcoxon rank sum test (continuous data) or chi-squared test (categorical data)/Fisher's exact test (categorical data with expected cell count < 5).

Neurodevelopmental outcomes were compared between children without CP and GDD from both the NE and non-NE group to look for more subtle developmental delays (Table 3, Fig. 4). Significant differences were seen in median DQ (− 2·9(95% CI: − 5·1, − 0·7)) and for gross and fine motor function scores (Locomotor, − 8·2(95% CI: − 13·1, − 3·7) and Eye & Hand, − 4·5(95% CI: − 8·0–0·8)). Although the median HINE score was the same in the two groups, the range was considerably wider for NE survivors leading to a statistically significant difference in HINE distribution. Suboptimal HINE scores (67–74) were seen in two NE survivors (DQs 102 and 98) and two non-NE children (DQ's 82 and 86) without CP or GDD. All were walking at two years.

**Table 3:** Developmental and neurology outcomes in unimpaired neonatal encephalopathy (NE) survivors and unimpaired non-NE comparison cohort at two years.

	Encephalopathy cohort (n = 82)	Comparison cohort (n = 227)	Median difference (95% CI)^a^	p-value†
Developmental outcomes				
Locomotor	100·0 (81·4, 111·2)	107·4 (90·6, 122·1)	− 8·2 (− 13·1, − 3·7)	< 0·001
Personal Social	112·0 (102·2, 122·1)	112·3 (102·5, 124·9)	− 0·3 (− 4·5, 3·8)	0·89
Speech & hearing	87·1 (83·0, 90·7)	87·7 (83·2, 95·0)	− 1·4 (− 3·5, 0·5)	0·17
Eye & hand	93·4 (86·1, 101·5)	100·6 (87·5, 110·1)	− 4·5 (− 8·0, 0·8)	0·02
Performance	81·1 (76·0, 84·1)	81·6 (76·4, 85·1)	− 0·9 (− 2·4, 0·5)	0·21
Practical reasoning	91·5 (86·1, 101·0)	95·9 (88·1, 103·2)	− 2·3 (− 5·4, 0·7)	0·13
Global developmental quotient	95·3 (89·4, 101·2)	97·7 (91·4, 104·1)	− 2·9 (− 5·1, − 0·7)	0·01
Neurology				
Hammersmith Infant Neurological Examination score^b^	78·0 (range: 68·0, 78·0)	78·0 (range: 73·0, 78·0)	0 (0, 0)	< 0·001

'Unimpaired'=survivors without cerebral palsy or global developmental delay. Data shown are median (IQR) for continuous data and n (%) for categorical data.

a Generalised Hodges-Lehmann median differences for continuous data.

b Not done for 4 children in the comparison cohort.

† p-value from Wilcoxon rank sum test.

Childhood seizures occurred in 5·7% of non-NE children (none on regular medication) but were more common amongst NE survivors (12·9%, RR 2·3) with 27% on regular medication. No significant differences were seen between NE children without CP or GDD and non-NE children for childhood seizures (data not shown).

#### 3.1.3 Type of Neurodevelopmental Impairment Seen

Table 4 details the clinical characteristics of children with NDI (34 NE survivors, 3 non-NE). Spastic bilateral CP was the commonest type of CP (63·6%) and was frequently associated with dystonia, 10 children had identified seizures (most with CP) and nearly half had under-nutrition. Nine children with NDI were microcephalic (z-score < − 3), two-thirds with bilateral spastic CP and a third with GDD.

**Table 4:** Clinical characteristics of children with neurodevelopmental impairment at two years of age amongst survivors of neonatal encephalopathy (NE) and the comparison, non-NE cohort.

No	NE Sarnat [16] stage	Age when assessed (months)	HINE	DQ	Cerebral Palsy	GMFCS	Hearing/visual impairment	Childhood Seizures	OFC cm (z-score)	Weight-for-age (z score)	Walking by 2 years	Type of neurodevelopmental impairment
Encephalopathy survivors												
1	2	27·0	13	3·3	Yes	5	VI	Yes	39·0 (− 6·98)	− 3·33	No	Spastic bilateral CP, dystonia, microcephaly
2	3	27·3	45·5	17·9	Yes	3	None	Yes	40·0 (− 6·27)	− 4·14	Yes AT	Spastic bilateral CP, dystonia, microcephaly
3	2	27·0	30	8·3	Yes	5	VI	Yes	40·2 (− 6·11)	− 6·49	No	Spastic bilateral CP, dystonia, microcephaly
4	2	27·1	18·5	1·7	Yes	5	HI &VI	Yes	42·3 (− 4·59)	− 3·75	No	Spastic bilateral CP, mild dystonia, microcephaly
5	3	28·7	17·5	2·8	Yes	5	VI	No	42·0 (− 4·12)	− 3·92	No	Spastic bilateral CP, no dystonia, microcephaly
6	3	28·3	19	1·9	Yes	5	HI & VI	No	43·5 (− 3·80)	2·00	No	Spastic bilateral CP, no dystonia, microcephaly
7	3	29·6	24	5·7	Yes	5	None	Yes	45·2 (− 1·91)	− 4·38	No	Spastic bilateral CP with dystonia
8	3	29·6	45·5	31·8	Yes	4	None	No	43·8 (− 2·91)	− 3·32	No	Spastic bilateral CP with dystonia
9	2	29·5	42	38·6	Yes	3	None	No	47·0 (− 1·36)	− 2·27	Yes AT	Spastic bilateral CP with dystonia
10	2	28·8	48·5	49·7	Yes	3	None	Yes	50·2 (+ 1·71)	− 1·26	No	Spastic bilateral CP no dystonia
11	3	27·1	33·5	31·4	Yes	4	None	No	45·8 (− 1·28)	− 1·10	No	Spastic bilateral CP with dystonia
12	2	26·9	20·5	11·1	Yes	5	None	Yes	49·7 (1·52)	− 4·39	No	Spastic bilateral CP marked dystonia,
13	2	27·0	41	33·3	Yes	3	None	No	49·8 (1·58)	− 1·35	No	Spastic bilateral CP legs more than arms
14	3	27·4	12	2·1	Yes	5	None	No	45·0 (− 1·88)	− 3·94	No	Spastic bilateral CP no dystonia
15	2	27·7	46	50·9	Yes	4	None	No	47·0 (− 0·47)	− 1·67	No	Dystonic CP
16	2	27·0	44·5	28·9	Yes	4	None	No	49·0 (+ 1·01)	− 0·14	No	Dystonic CP
17	2	28·2	39	35·1	Yes	3	None	No	47·8 (+ 0·05)	− 2·78	Yes	Choreo-athetotic CP
18	2	27·2	64	78·8	Yes	2	None	No	49·5 (+ 1·35)	1·54	Yes	Unilateral CP, left hemiplegia
19	3	27·4	51	75·9	Yes	3	None	No	47·0 (− 0·45)	− 0·24	No	Bilateral CP, diplegia
20	2	29·7	61·5	83·5	Yes	1	None	No	49·5 (1·15)	− 1·30	Yes	Bilateral CP, diplegia
21	2	29·8	70	30·9	Yes	3	None	Yes	44·0 (− 1·29)	− 3·34	No	Unclassifiable CP, global hypotonia
22	1	27·8	62	53·6	Yes	2	None	No	46·4 (− 1·67)	− 2·57	Yes	Unclassifiable CP, global hypotonia
23	2	28·9	71	36·4	No	0	None	No	42·0 (− 4·14)	− 3·84	Yes	Global developmental delay, microcephaly
24	3	28·1	71	66·2	No	0	None	No	43·0 (− 3·36)	− 4·38	Yes	Global developmental delay, microcephaly
25	2	28·4	77	61·3	No	0	None	No	44·5 (− 3·08)	− 1·50	Yes	Global developmental delay, microcephaly
26	2	27·3	73	41·8	No	0	None	Yes	46·9 (− 0·51)	− 1·26	Yes AT	Global developmental delay
27	2	27·2	74	52·2	No	0	None	No	47·5 (− 0·07)	0·30	Yes	Global developmental delay
28	2	27·3	78	66·3	No	0	None	No	47·5 (− 0·09)	0·11	Yes	Global developmental delay
29	2	27·9	77	65·9	No	0	None	No	47·8 (+ 0·08)	− 0·10	Yes AT	Global developmental delay
30	2	27·6	76	67·8	No	0	None	No	48·5 (+ 0·61)	− 0·70	Yes	Global developmental delay
31	1	28·3	78	58·7	No	0	None	No	50·5 (+ 1·97)	0·13	Yes	Global developmental delay
32	2	28·1	76·5	56·2	No	0	None	No	48·2 (− 0·39)	− 0·39	Yes	Global developmental delay
33	2	28·1	78	61·6	No	0	None	Yes	44·4 (− 2·36)	0·03	Yes	Global developmental delay
34	3	27·0	76	55·9	No	0	None	No	45·5 (− 1·49)	− 0·58	Yes	Global developmental delay

Comparison cohort												
1	0	28·6	67·5	72·0	Yes	2	None	No	47·0 (− 1·29)	− 2·11	Yes AT	Bilateral CP, mild diplegia
2	0	27·5	58·5	72·4	Yes	1	None	No	48·0 (+ 0·25)	− 1·23	Yes	Dystonic CP
3	0	29·3	78	51·4	No	0	None	No	46·8 (− 0·75)	− 0·70	Yes	Global developmental delay

CP = Cerebral Palsy; DQ, Developmental quotient; GMFCS, Gross Motor Function Classification System; HI = hearing impairment; HINE = Hammersmith Infant Neurological Examination; OFC, Occipito-frontal circumference; VI = visual impairment; AT = with assistive technology.

All non-NE children were ambulant and none microcephalic, but two had CP (one mildly diplegic and one dystonic) and low DQs. Another child had GDD without motor impairment and one had profound hearing loss but DQ and HINE scores within normal range. All children with GMFCS scores 3–5 (moderate-to-severe cerebral palsy) had low HINE scores and all those with global developmental disability but no cerebral palsy had scores > 67. The underlying aetiologies were unclear.

#### 3.1.4 Nutrition and Other Health Outcomes in NE Survivors

NE survivors were significantly more likely to have moderate (RR1·9) or severe (RR7·9) under-nutrition compared to non-NE children (Table 2). This increased risk was driven by those with NDI, (weight-for-age Z-score < − 2, 44·1% in impaired vs. 3·7% unimpaired, RR12·1(3·7–39·0) and height-for-age Z-score < − 2, 5·9% in impaired vs.18·3% in unimpaired, RR2·9(1·7–5·0)). A third of NE children with NDI (35·3%) had severe undernutrition (weight-for-age Z-score < − 3) versus none without NDI.

Mean Hb levels were slightly lower in NE survivors, however, the prevalence of anaemia was similar. Chest infections were more common amongst children with NE than in the non-NE group (22/116(19·0%) and 18/230(7·8%) respectively, p = 0·002) and amongst NE survivors with NDI than those without NDI (10/34(29·4%) and 12/82(14·6%), p = 0·07).

#### 3.1.5 Mortality After NE

**Fig. 3:**
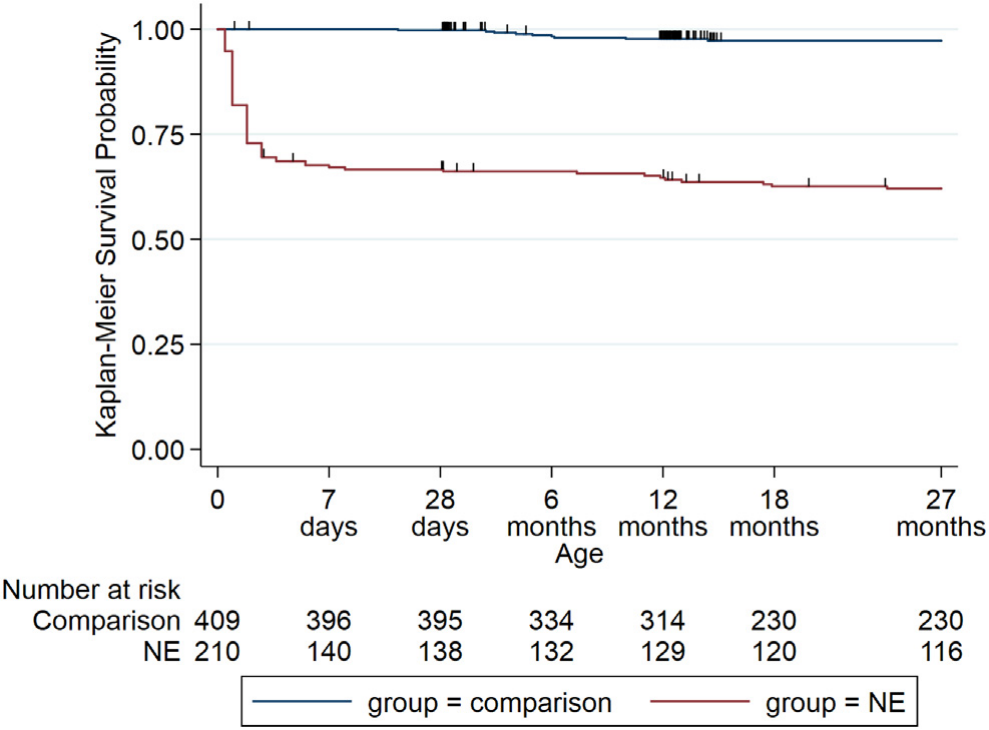
Survival estimates for those with and without neonatal encephalopathy during the neonatal period.

Neonatal fatality amongst the encephalopathy and non-NE cohorts has been reported previously [12]. In the encephalopathy cohort, most deaths, 91·4% (64/70), occurred before day 4 and 98·6% (69/70) before day 8 (Fig. 3). Mortality in the NE and non-NE cohorts are shown in Table 5.

**Table 5:** Mortality in the neonatal encephalopathy (NE) and comparison, non-NE cohorts.

Follow-up period	Encephalopathy cohort^a^	Comparison cohort^a^	p-value (chi squared test)	p-value (log rank test)
Neonatal	70/208 (33.7%)	1/396 (0.3%)	< 0.001	< 0.001
Post-neonatal	11/127 (8.4%)	8/238 (3.4%)	0.03	0.008
Birth to 27 months	81/201 (40.3%)	9/239 (3.8%)	< 0.001	< 0.001

a Numerator = number of deaths during follow-up period, denominator = number of participants for whom vital status known at end of follow-up period.

**Fig. 4:**
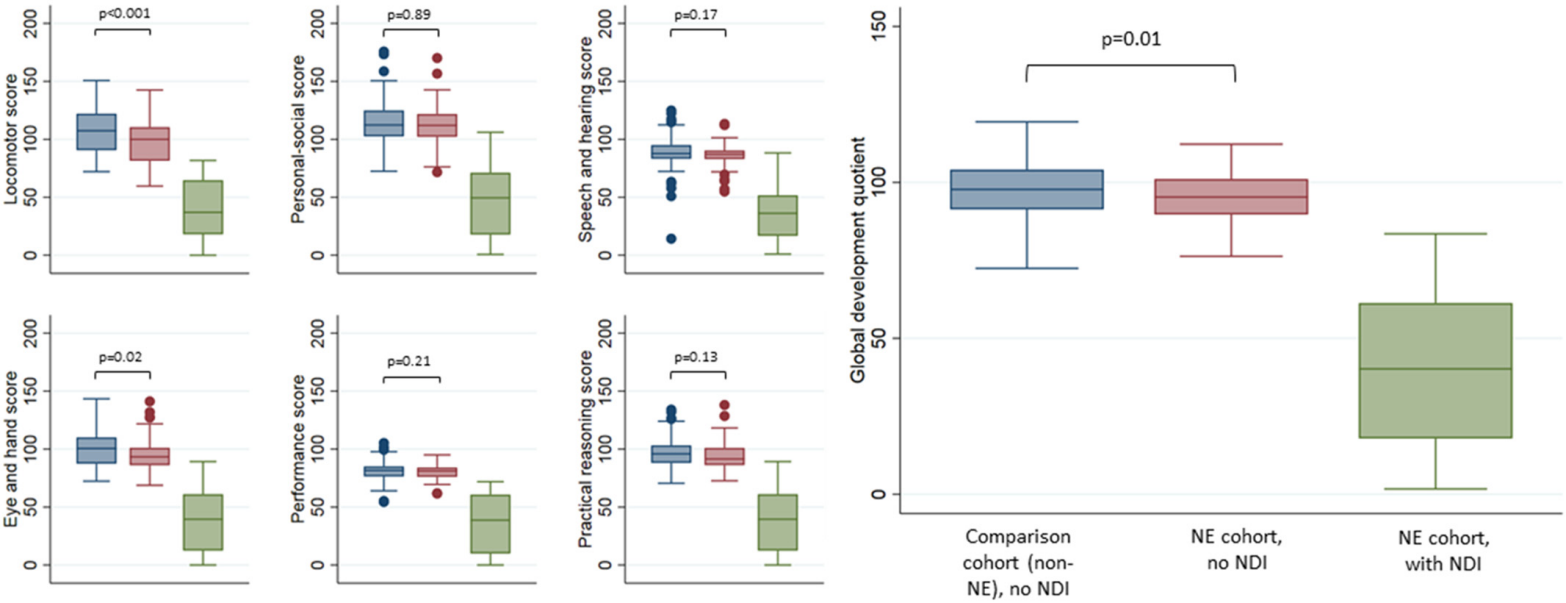
Griffiths Mental Development Scales global developmental quotient (DQ) and six-subscale scores of comparison group, NE survivors without impairment, and those with impairment. The blue, red and green bars represent comparison cohort without neonatal encephalopathy (non-NE) with no NDI, neonatal encephalopathy (NE) cohort with no NDI, and NE cohort with NDI, respectively. P-values for comparison between NE cohort with NDI and the other two groups all < 0.001.

In the NE cohort, six children died in early infancy (two moderate and four severe NE). Causes of death (parental report) included pneumonia(n = 2), seizures(n = 2), diarrhoea and vomiting(n = 1) and unknown(n = 1). A further five children died between 12 and 27 months. Four had been assessed at 12 months to have severe four-limb CP following severe NE. Cause of death was attributed to seizures(n = 2), pneumonia(n = 1) and pneumonia/malaria(n = 1). The fifth child had CP with severe motor delay but otherwise normal development following moderate NE; the cause of death was given as pneumonia. Amongst the non-NE group, seven infants died in the first fifteen months (pneumonia/febrile illness(n = 5), cardiac condition(n = 1), motorcycle accident(n = 1), unknown(n = 1).

#### 3.1.6 Clinical Predictors of Death and Disability After NE

The risk of an adverse outcome (death or NDI) significantly increased with NE severity [16]. Death or NDI occurred in 21·7%(5/23) with mild, 50·9%(54/106) with moderate and 82·4%(56/68) were severe NE (p < 0·0001, trend). Similarly, with the Thompson score, 37·5% (30/80) with score 6–10, 68·8%(66/96) with score 11–14, and 90·5%(19/21) with score 15–22 had an adverse outcome (p < 0·0001, trend). Adverse outcome was more common amongst those with endogenous hypothermia on day 1 (60·9% with hypothermia vs. 48·7% without, RR1·25(0·88–1·78)). No significant outcome differences were seen between those with and without proven neonatal serious bacterial infection; 68·6%(11/16) with bacteraemia vs. 57·5%(104/181) without (p = 0·38). cUS abnormality (in BGT and/or WM) on day 4/5 was strongly associated with increased risk of adverse outcome (39/47(83·0%) for abnormal scans versus 6/46(13·0%) for normal scans), RR6·4(3·0, 13·6)).

The risk of NDI amongst survivors increased significantly with NE severity (Table 6), neonatal clinical seizures, an abnormal cUS on day 4/5 and nasogastric feeding at discharge but not day 1 endogenous hypothermia (Table 6). All children with OFC z-score < − 2 at one year had NDI at 27–30 months.

**Table 6:** Clinical predictors of impairment at two years in neonatal encephalopathy survivors.

Clinical predictor		NDI N (%)	RR (95% CI)	p-value
Severity of encephalopathy: Sarnat staging [16]				
Mild		2/20 (10·0%)	1	0·04
Moderate		22/74 (29·7%)	3·0 (0·8, 11·6)	
Severe		10/22 (45·5%)	4·6 (1·1, 18·3)	
Severity of encephalopathy: Thompson score [13]				
6–10		13/63 (20·6%)	1	0·01
11–14		16/46 (34·8%)	6·3 (3·4, 11·8)	
15–22		5/7 (71·4%)	13·0 (6·7, 25·3)	
Neonatal clinical seizures				
No		7/60 (11.7%)	1	
Yes		27/56 (48.2%)	4.1 (2.0, 8.7)	< 0·001
Hypothermia day 1 (axillary temperature < 36·5C)				
No		7/26 (26·9%)	1	
Yes		27/88 (30·7%)	1·1 (0·6, 2·3)	0·71
Cranial ultrasound abnormality on early (day 1) scan				
No abnormality		29/90 (32·2%)	1	
Abnormality		5/13 (38·5%)	1·2 (0·6, 2·5)	0·66
Cranial ultrasound abnormality on late (day 4/5) scan				
No abnormality		5/45 (11·1%)	1	
Abnormality		28/36 (77·8%)	7·0 (3·8, 16·3)	< 0·001
Nasogastric feeding on discharge				
No		15/85 (17·7%)	1	
Yes		19/30 (63·3%)	3·6 (2·1, 6·1)	< 0·001
Occipito-frontal head circumference at one year of age^a^				
Z-score < − 2 (< 41·8 cm)	No	27/109 (24·8%)	1	
	Yes	7/7 (100·0%)	4·9 (2·9, 5·6)	< 0·001
Z-score < − 3 (< 39·5 cm)	No	32/114 (28·1%)	1	
	Yes	2/2 (100·0%)	3·6 (2·7, 4·8)	0·03

RR = Risk Ratio; CI = Confidence Intervals.

a Cut-offs for occipito-frontal head circumference was determined as comparison group mean – 2 comparison group SD and mean – 3 comparison SD.

## 4 Discussion

We report here on survival, developmental, and nutritional outcomes of children after NE in a sub-Saharan African setting [2], [4]. Our large NE and comparison cohorts, low loss-to follow-up rates and comprehensive neurodevelopmental assessments allow us to give accurate early childhood outcomes after NE in this urban Ugandan population. A third of survivors were affected by neurodevelopmental impairments. Bilateral spastic CP, frequently with dystonia was common and strongly associated with multi-domain impairment. Several clinical parameters strongly predicted adverse outcome at two including severity of NE, presence of neonatal seizures, abnormalities on cranial ultrasound, persistent neonatal feeding difficulties and a small head circumference at one year. Most deaths after NE occurred in the neonatal period and post-neonatal mortality high amongst children identified to have CP.

In our sub-Saharan African study, 2-year disability-free survival occurred in 41·6% of NE survivors. This is not dissimilar to studies from high-income country settings; disability-free survival was 46·9% amongst the control arm of the Toby therapeutic hypothermia trial (UK) [27] and 39% in Western Australia [3] although differing definitions of NE and differing survival rates affect study comparability. In our setting, a high proportion of NE survivors had moderate or severe NDI at 2 years compared to normal term-born children. Severe, bilateral spastic CP was the most common condition, followed by severe GDD without CP. Hearing and visual impairment only occurred with NDI, but may be underestimated since neurophysiological assessments could not be performed.

Malnutrition was common amongst those with NDI, of whom nearly half had moderate or severe undernutrition and/or stunting. This likely reflects feeding difficulties associated with severe CP as well as increased use of calories. Assessing nutritional status amongst children with neurodisability presents challenges [28] but maintaining good nutrition is crucial to maximising their health, functioning and quality of life [29].

Important differences were also seen in neurodevelopmental outcome after excluding children with CP and GDD from both cohorts. As in the Western Australia study [3], children who had NE but did not develop CP or GDD were still at increased risk of poorer motoring function compared to children without NE. Previous studies have shown motor problems in children without CP after HIE [30]. Published data on later childhood outcomes after NE in LMICs is lacking. Longer-term follow-up of our cohort is needed to assess differences in cognitive and behavioural outcomes likely unapparent at this early age.

The highest risk of death after NE was seen in the early neonatal period, consistent with other studies from LMICs [25]. This increased risk continued however through the first two years with NE survivors at three times the risk of post-neonatal death compared to the non-NE cohort. In NE survivors most deaths were related to severe, four-limb spastic CP with deaths from pneumonia and childhood seizures the most common. The substantial contribution of term-age neonatal illness to the aetiology of CP in this urban Ugandan setting has been reported by us previously [26]. Since studies examining the aetiology of CP commonly use a 2-year age cut-off, high early childhood mortality rates amongst impaired NE survivors may lead to under-estimation of the contribution of NE to childhood disability in this setting.

We identified simple clinical predictors of NDI after NE including clinical severity of NE, presence of neonatal clinical seizures, the need for continued feeding support and small head circumference at one year. Identification of simple, early clinical predictors of outcome has the potential to support targeted neurodevelopmental follow-up in those at highest risk in low resource settings. It was not our intention to report on the sequential development of children within this paper, although it would be valid and of interest to do so. We have retained our focus on two-year outcomes when cerebral palsy can be more reliably diagnosed and assessment below this age may miss impairment. Short cohort follow-up has been a major limitation of previous studies in this field.

#### 4.1.1 Strengths and Limitations

We achieved high follow-up rates amongst NE survivors and lower rates amongst the non-NE cohort, with some baseline socio-demographic differences between those who were and were not followed which could lead to bias when comparing the NE and non-NE cohorts. The diagnosis of childhood seizures and chest infections was taken from parent handheld records, verified by clinician report however reporting bias cannot be excluded. Risk of observer bias was minimised; all children being assessed by examiners blind to NE status and clinical data. All children were videoed, and those with suboptimal scoring reviewed to ensure correct diagnosis and typing of impairment. A comparison cohort of term children without NE allowed comparison of GMDS and HINE scores, as standardised Ugandan data for these tests is not available. The lack of a standardised definition of NE limits the comparability of data between countries and resource settings. A large number of statistical tests were undertaken; however, our results are consistent, both internally and externally, thus our findings are unlikely to be due to multiplicity.

## 5 Conclusion

Death and disability after NE at two years of age are common in this low resource sub-Saharan African setting. Severe, four-limb CP is the most common disability, and this is often associated with global developmental problems, malnutrition and seizure disorders. The high burden of early mortality amongst children with severe impairment in this setting means that the contribution of NE to childhood disability may be under-estimated. This study has identified simple clinical parameters that may facilitate targeted follow-up of infants at greatest risk. Children without overt impairment had an increased risk of delay in motor functioning. It is important to undertake longer-term follow-up to determine whether further problem emerge as educational, motor and social demands increase. Strategies for the prevention of NE, and early intervention for those affected by neurodisability, are urgently needed in LMICs to maximise functional and nutritional outcomes and quality of life for affected children and their caregivers.

The following is the supplementary data related to this article.

Supplementary Table Characteristics of comparison cohort children seen and not seen at 27–30 months (excluding known deaths). Supplementary Table

## Contributors

CT, JK, NR, AE, FC and MN contributed to the conception and design of the original and follow-up studies and MMB to the design of the cohort follow-up. All authors contributed to interpretation of the data. CT implemented and led the study and RL, JS, JM, KN, EDB, MM, MS, MMB and KB participated in acquisition of data including all clinical and neurodevelopmental assessments. Cranial ultrasound scan images were reported by FC and CH. Data analysis was conducted by EW and CT. CT, EW and FC wrote the first draft of the paper. All authors reviewed, critically revised and approved the final version of the manuscript and agree to be accountable for all aspects of the work.

## Declaration of Interests

We declare no competing interests.

## References

[1] Lawn J.E., Blencowe H., Oza S. (2014). Every newborn: progress, priorities, and potential beyond survival. Lancet.

[2] Lee A.C., Kozuki N., Blencowe H. (2013). Intrapartum-related neonatal encephalopathy incidence and impairment at regional and global levels for 2010 with trends from 1990. Pediatr Res.

[3] Dixon G., Badawi N., Kurinczuk J.J. (2002). Early developmental outcomes after newborn encephalopathy. Pediatrics.

[4] Mwaniki M.K., Atieno M., Lawn J.E., Newton C.R. (2012). Long-term neurodevelopmental outcomes after intrauterine and neonatal insults: a systematic review. Lancet.

[5] United Nations Children's Fund Early Child Devlopment Unit (2006). Programming experiences in early Child development.

[6] World Health Organisation Disability, Rehabilitation Team (2011). World report on disability rehabilitation. Geneva.

[7] Kuruvilla S., Bustreo F., Kuo T. (2016). The global strategy for women's, children's and adolescents' health (2016 − 2030): a roadmap based on evidence and country experience. Bull World Health Organ.

[8] Ouellette-Kunz H. (2005). Understanding health disparities and inequities faced by individuals with intellectual disabilities. J Appl Res Intellect Disabil.

[9] Nakamanya S., Siu G.E., Lassman R., Seeley J., Tann C.J. (2015). Maternal experiences of caring for an infant with neurological impairment after neonatal encephalopathy in Uganda: a qualitative study. Disabil Rehabil.

[10] Sharma R., Gaffey M.F., Alderman H. (2017). Prioritizing research for integrated implementation of early childhood development and maternal, newborn, child and adolescent health and nutrition platforms. J Glob Health.

[11] Uganda Demographic and Health Survey (2016). Kampala, Uganda & Maryland.

[12] Tann C.J., Nakakeeto M., Willey B.A. (2018 May). Perinatal risk factors for neonatal encephalopathy: an unmatched case-control study. Arch Dis Child Fetal Neonatal Ed.

[13] Thompson C.M., Puterman A.S., Linley L.L. (1997). The value of a scoring system for hypoxic ischaemic encephalopathy in predicting neurodevelopmental outcome. Acta Paediatr.

[14] Tann C.J., Nkurunziza P., Nakakeeto M. (2014). Prevalence of bloodstream pathogens is higher in neonatal encephalopathy cases vs. controls using a novel panel of real-time PCR assays. PLoS One.

[15] Tann C.J., Nakakeeto M., Hagmann C. (2016). Early cranial ultrasound findings among infants with neonatal encephalopathy in Uganda: an observational study. Pediatr Res.

[16] Sarnat H.B., Sarnat M.S. (1976). Neonatal encephalopathy following fetal distress. A clinical and electroencephalographic study. Arch Neurol.

[17] Luiz D., Barnard A., Knoesen N. (2006). GMDS-ER: Griffiths Mental Development Scales – Extended revised: Two to eight years: Administration manual.

[18] Romeo D.M., Ricci D., Brogna C., Mercuri E. (2016). Use of the Hammersmith infant neurological examination in infants with cerebral palsy: a critical review of the literature. Dev Med Child Neurol.

[19] Bosanquet M., Copeland L., Ware R., Boyd R. (2013). A systematic review of tests to predict cerebral palsy in young children. Dev Med Child Neurol.

[20] Haataja L., Mercuri E., Guzzetta A. (2001). Neurologic examination in infants with hypoxic-ischemic encephalopathy at age 9 to 14 months: use of optimality scores and correlation with magnetic resonance imaging findings. J Pediatr.

[21] (2000). Dev Med Child Neurol.

[22] Gorter J., Ketelaar M., Rosenbaum P., Helders P., Palisano R. (2009). Use of the GMFCS in infants with CP: the need for reclassification at age 2 years or older. Dev Med Child Neurol.

[23] (2009). WHO child growth standards and the identification of severe acute malnutrition in infants and children: A joint statement by the World Health Organization and the United Nations Children's fund.

[24] WHO Multicentre Growth Reference Study Group (2006). WHO Child Growth Standards based on length/height, weight and age. Acta Paediatr Suppl.

[25] Ellis M., Manandhar N., Shrestha P.S., Shrestha L., Manandhar D.S., Costello A.M. (1999). Outcome at 1 year of neonatal encephalopathy in Kathmandu, Nepal. Dev Med Child Neurol.

[26] Hassell J., Tann C.J., Idro R., Robertson N.J. (2018). Contribution of perinatal conditions to cerebral palsy in Uganda. Lancet Glob Health.

[27] Azzopardi D., Strohm B., Edwards A.D. (2009). Treatment of asphyxiated newborns with moderate hypothermia in routine clinical practice: how cooling is managed in the UK outside a clinical trial. Arch Dis Child Fetal Neonatal Ed.

[28] Gladstone M., Mallewa M., Alusine Jalloh A. (2014). Assessment of neurodisability and malnutrition in children in Africa. Semin Pediatr Neurol.

[29] Martinez-Biarge M., Diez-Sebastian J., Wusthoff C.J. (2012). Feeding and communication impairments in infants with central grey matter lesions following perinatal hypoxic-ischaemic injury. Euro J Paediatr Neurol.

[30] van Kooij B.J., van Handel M., Nievelstein R.A., Groenendaal F., Jongmans M.J., de Vries L.S. (2010). Serial MRI and neurodevelopmental outcome in 9- to 10-year-old children with neonatal encephalopathy. J Pediatr.

