# Supplementary material for: Early Childhood Outcomes After Neonatal Encephalopathy in Uganda: A Cohort Study
Source: eClinicalMedicine. 2018 Dec 20;6:26–35. doi: 10.1016/j.eclinm.2018.12.001 (PMC6358042; doi:10.1016/j.eclinm.2018.12.001)
Supplement: Supplementary Table — Characteristics of comparison cohort children seen and not seen at 27–30 months (excluding known deaths). [file mmc1.docx]

**Supplementary Table. Characteristics of comparison cohort children seen and not seen at 27-30 months (excluding known deaths)**

| **Characteristic** | **Comparison cohort members seen at 27-30 months (N=230)**  **n (%)** | **Comparison cohort members not seen at 27-30 months (N=170)**  **n (%)** | **p-value*** |
| --- | --- | --- | --- |
| **Maternal Factors** |  |  |  |
| Socio-economic group High | 49 (21.7%) | 30 (17.7%) | 0.002 |
| Medium | 147 (65.0%) | 93 (54.7%) |  |
| Low | 30 (13.3%) | 47 (27.7%) |  |
| Maternal age in years, mean (SD) | 24.9 (5.4) | 22.7 (4.6) | <0.001 |
| Maternal education ≤primary school | 84 (36.7%) | 62 (36.5%) | 0.97 |
| Maternal primaparity | 84 (36.5%) | 95 (55.9%) | <0.001 |
| Maternal HIV positive | 27 (11.7%) | 23 (13.5%) | 0.59 |
| Emergency caesarean section | 38 (16.5%) | 17 (10.0%) | 0.06 |
| **Infant Factors** |  |  |  |
| Male sex | 114 (49.6%) | 76 (44.7%) | 0.34 |
| Birth weight in kg, mean (SD) | 3.16 (0.46) | 3.06 (0.40) | 0.02 |
| Birth occipito-frontal head circumference in cm, mean (SD) | 34.9 (1.7) | 34.6 (1.8) | 0.10 |
| **Postnatal factors** |  |  |  |
| Apgar score at 5 minutes ≤5 | 0 (0%) | 1 (0.6%) | 0.42 |
| Need for any resuscitation | 33 (15.1%) | 23 (15.1%) | 0.99 |
| **Clinical features (day 1)** |  |  |  |
| Hypothermia: axillary temperature <36.5C^†^ | 52 (23.9%) | 56 (35.7%) | 0.01 |
| Hyperthermia: axillary temperature >37.5C^†^ | 4 (1.8%) | 4 (2.5%) | 0.46 |
| Haemoglobin in g/L, mean (SD) ^†^ | 18.1 (2.0) | 18.2 (2.6) | 0.52 |

Missing data for SES (4 seen), maternal age (1 not seen), maternal education (1 seen), occipito-frontal head circumference (2 seen, 7 not seen), Apgar scores (3 seen, 7 not seen), resuscitation (11 seen, 18 not seen), temperature (12 seen, 13 not seen), haemoglobin (30 seen, 25 not seen). ^*^p-values calculated using chi-squared or Fisher’s exact tests for categorical data and t-tests for continuous data. ^†^Temperature and haemoglobin measured during day 1
